# The magnitude of standard precautions practice and its associated factors among healthcare workers in governmental hospitals of northeastern Ethiopia

**DOI:** 10.3389/frhs.2023.1071517

**Published:** 2023-03-22

**Authors:** Awoke Keleb, Mistir Lingerew, Ayechew Ademas, Gete Berihun, Tadesse Sisay, Metadel Adane

**Affiliations:** Department of Environmental Health, College of Medicine and Health Sciences, Wollo University, Dessie, Ethiopia

**Keywords:** associated factors, guideline adherence, healthcare, practice, standard precaution

## Abstract

**Background:**

Non-compliance with infection control guidelines of healthcare workers may increase their risk of exposure to infectious diseases but can be prevented through adherence to standard precautionary practices in healthcare settings.

**Objective:**

This study aimed to assess the magnitude of standard precautions practice and its associated factors among healthcare workers in government hospitals of South Wollo Zone, northeastern Ethiopia

**Methods:**

An institutional-based cross-sectional study was conducted among 1,100 healthcare workers. Proportional sample size allocation for each selected government hospital was conducted followed by simple random sampling to select study participants using human resource records from each hospital. Data were collected using structured and self-administered pretested questionnaires. The data were analyzed using descriptive statistics, bivariable binary, and multivariable logistic regression models. Variables with a *p*-value <0.05 with a 95% CI were considered as having statistical significance

**Results:**

The overall magnitude of compliance with standard precautions among healthcare workers was 19.2%. The result indicated that work experience of <5 years (AOR = 2.51; 95% CI: 1.07–5.89), absence of continuous water supply (AOR = 2.24; 95% CI: 1.95–5.29), and negative attitude (AOR = 2.37; 95% CI: 1.17–4.79) were significantly associated with poor compliance of standard precautions practice.

**Conclusion:**

The overall magnitude of compliance with standard precautions among healthcare workers was low compared to the national magnitude of infection prevention practice. Interventions including consistent and effective training on infection prevention healthcare workers should be given regularly. Providing continuous water supply and building a positive attitude toward infection prevention practices among healthcare workers are also required.

## Introduction

Standard precautions (SP) also known as universal precautions are the primary method for securing worker safety and preventing Health Care Associated Infections (HCAI) including the COVID-19 pandemic. Evidence from national and international literature indicates that few professionals properly applied these guidelines (1). Various reasons contribute to the behavior of non-adherence, including institutional elements like material, human resources, and managerial commitment as well as individual factors like understanding of the SP measures (2, 3).

Hospitals are the main source of infection risk when delivering healthcare services. HCAIs are frequently disseminated by contact, airborne transmission, and droplets. They can also transmit between healthy people and the infected person, especially during hospitalization (4, 5). A significant portion of occurrences of HCAI is linked to workplace exposures (4, 6). The health and well-being of our healthcare workers and the public at large determine the nation's health, security, and economic progress in Ethiopia.

According to the World Health Organization (WHO), HCAI or “nosocomial” and “hospital” infections are the most frequent adverse event in healthcare delivery worldwide. Those infections are acquired in hospitals or healthcare service units, that first appear 48 h or more after hospital admission or within 30 days after discharge following in-patient care. They are a significant public health issue that raises morbidity, mortality, and financial losses for healthcare systems (4).

Around 3 million healthcare workers are exposed to bloodborne infections through percutaneous means each year globally of which two million of these workers are exposed to the hepatitis B virus (HBV), about one million to the hepatitis C virus (HCV), and 170,000 to the human immunodeficiency virus (7).

According to a systematic review and meta-analysis on the burden of HCAIs in Southeast Asia, the pooled prevalence of all HAIs was 9.0%, while the combined incidence density was 20 cases per 1,000 intensive care units (8). About 7 patients in industrialized countries and 10 individuals in poor nations out of every 100 hospitalized patients at any given moment acquire at least one HCAI. It affects 5% to 15% of hospitalized patients in ordinary wards and up to 50% or more of patients in intensive care units in developed nations (9).

In low and middle-income countries the frequency of intensive care unit-acquired infections is at least 2–3 times higher than in high-income countries, and healthcare-associated infection densities might be up to 13 times greater than in the USA (4, 10). Yet the size of the issue is currently underappreciated or even unknown, largely due to the complexity of HCAI diagnosis and the need for knowledge and resources for surveillance operations to inform solutions (11).

However, it was discovered in a meta-analysis to evaluate the burden of HCAI in poor nations that the proportion of HCAI was substantially higher (15•5 per 100 patients) than proportions reported from Europe and the USA (12). HCAIs account for 4% to 56% of all neonatal period deaths among hospital-born infants in underdeveloped countries, and 75% in South-East Asia and Sub-Saharan Africa (11, 12). The prevalence of healthcare-associated infections in teaching hospitals of Ethiopia's Amhara region was 14.9% (13).

Local reports revealed that there is still a high incidence of HCAIs despite a major development in the infrastructure of health institutions and the number and kind of health task forces, especially in Ethiopia, during the past 20 years (13, 14).

Healthcare-associated infections cause additional pain and have a significant financial impact on patients and their families, as is the case with many other patient safety issues. Infections prolong hospital stays, create long-term disability, increase resistance to antimicrobials, represent a massive additional financial burden for health systems, generate high costs for patients and their families, and cause unnecessary deaths.

To prevent infections during patient care and invasive operations, the implementation of fundamental standard precautions is easy and inexpensive, but it necessitates staff accountability and behavioral change (4, 6, 15). To improve the safety of patients, visitors, attendants, HCWs, and the general community in healthcare facilities, WHO launched different programs like “clean care is safe care” as a key strategy to promote infection prevention practice at all levels of healthcare settings (5, 16).

To prevent potential ease of transmission and thereby the illness, routine standard precautions are also advised by the WHO and other national and international public health organizations (17, 18). Healthcare regulations are not always followed in many healthcare institutions, and this indicates that compliance should be routinely examined to determine the elements that may help or hinder it. However, there is a large disparity in the level of compliance with standard precautions (5).

Due to the rising need for modern healthcare, Ethiopia is experiencing a significant increase in the number of healthcare facilities, so the government has implemented favorable laws to promote the establishment of both public and private hospitals. Even though the northeastern portions of Ethiopia have many hospitals and a considerable number of healthcare professionals, the state of compliance with standard precaution procedures and its determinant factors remains unexplored.

Due to the lack of scientific evidence, two obvious research questions arise:

Firstly, what is the level of compliance with standard precautions measures? and secondly, what are the predictors of inadequate adherence to standard precautionary measures?

The results of this study would help policymakers and planners to create effective intervention and control strategies for healthcare-acquired infections. Therefore, the purpose of this study was to evaluate how well healthcare professionals in public hospitals in the South Wollo zone in northeastern Ethiopia complied with standard precautions practice and the associated factors.

## Methods

### Study area

**Figure 1:**
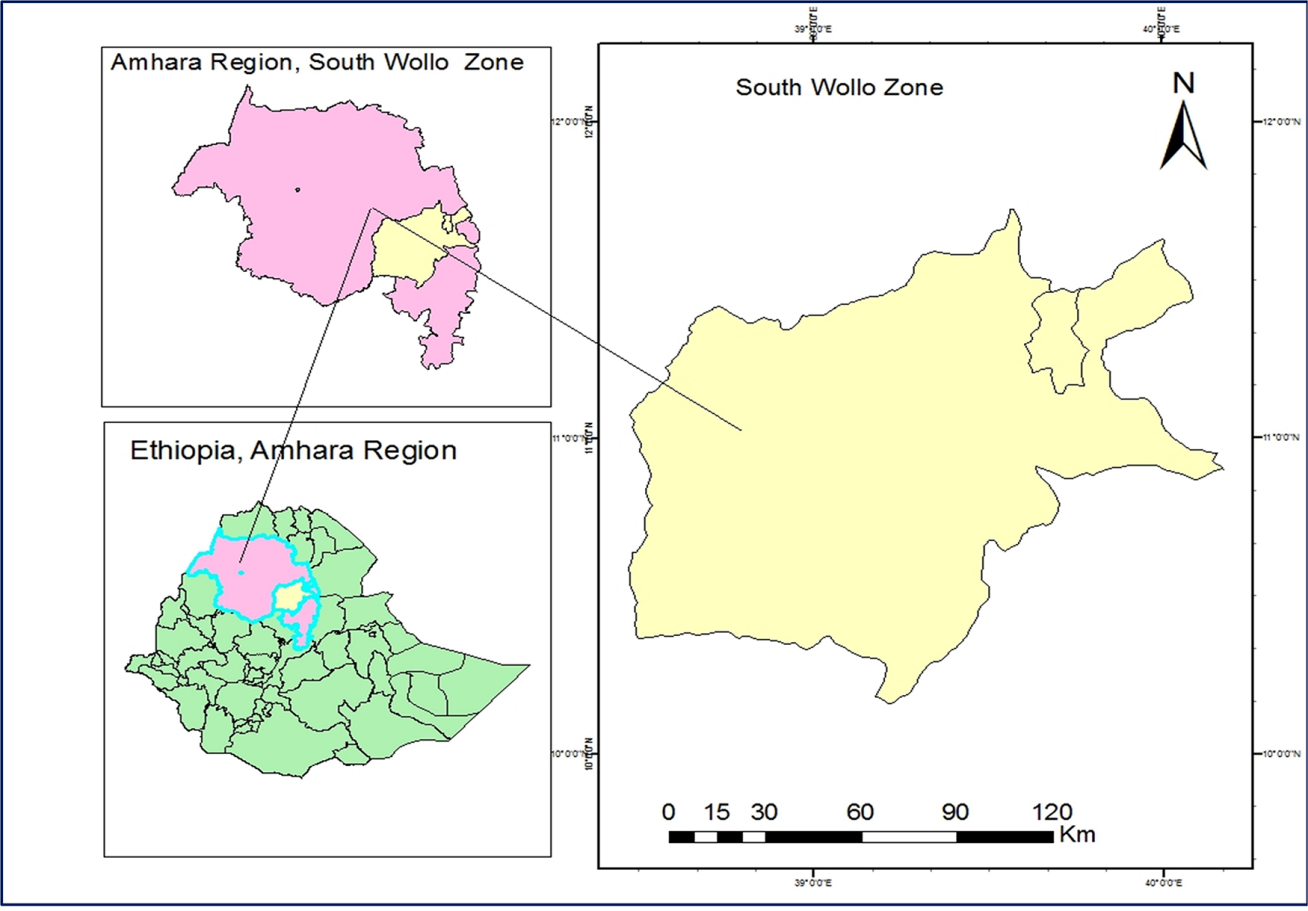
Map of study area.

South Wollo is one of ten zones in the Amhara Region of Ethiopia. South Wollo is bordered on the south by North Shewa and the Oromia Region, on the west by West Gojjam, on the northwest by South Gondar, on the north by North Wollo, on the northeast by Afar Region, and on the east by the Oromia Zone and the Argobba special woreda (Figure 1).

South Wollo Zone has eleven (11) hospitals with a catchment population of 2,518,862. Of which 301,638 (11.98%) are urban inhabitants, a further 2,217.224 (88.02%) inhabitants were reported to be rural; it also has an area of 17,067.45 square kilometers, with a population density of 147.58.

### Study design and period

An institutional-based cross-sectional study was conducted to assess the magnitude of compliance with standard precautions practice and its associated factors among healthcare workers in public hospitals of South Wollo zone in northeastern Ethiopia from June to July 2021.

### Source population and inclusion criteria

In this study, healthcare workers were defined as regularly employed hospital staff who provided direct patient care or had regular direct contact with patients, as well as graduating class intern doctors. The source population of this study was all healthcare workers working in hospitals while the study population was all selected healthcare workers in hospitals of South Wollo zone in northeastern Ethiopia. From the study population, all permanent healthcare workers employed in the hospitals were included in the study.

### Sample size determination and sampling methods

The sample size was determined using the single population proportion formula with the following assumptions: Magnitude of compliance with standard precaution practice (*p* = 12%) was taken from a study conducted in Gondar University comprehensive specialized hospital, northwest Ethiopia (19)

$$n=\frac{{\left(z_{a/2}\right)}^{2}\;*\;p(1-p)}{d^{2}}$$

(20) Where: ***n***: is the optimum sample size required.

***P***: is an estimate of the magnitude of compliance with standard precaution.
***Z***: is the standard normal variable at (1-*α*) % confidence level and *α* is mostly 0.05 i.e. with 95% CI (*z* = 1.96) and **d**: is the margin of error to be tolerated (%).

The determination of the margin of error is based on the optimum sample size and availability of resources.1% giving the largest sample size and 5% giving the smallest sample size. For this study, it was used a margin of 2% based on the proportion of 12% taken from a similar study mentioned above, which gives an adequate sample size.

$$n=\frac{{\left(1.96\right)}^{2}\;*\;0.12(1-0.12)}{{\left(0.02\right)}^{2}}=1014$$

After adding 10% non-response rate the final sample size was ***n* =** **102 + 1014 = 1116**.

There are eleven hospitals in the South Wollo zone from which four hospitals were selected randomly. All 1,116 estimated participants were proportionally allocated to each hospital*-*based upon their respective numbers of healthcare workers. The study participants were selected using a simple random sampling method. Those study participants that were not available at the first visit, the study participants were revisited once the same day or following day. Those who were not available again, the study participants were considered as a non-respondent.

$$\begin{array}{rl} & \mathrm{Sample}\;\mathrm{size}\;\mathrm{for}\;\mathrm{each}\;\mathrm{hospital}=\\ & \frac{\mathrm{No}\;\mathrm{of}\;\mathrm{healthcare}\;\mathrm{workers}\;\mathrm{per}\;\mathrm{hospital}\;*\;\mathrm{total}\;\mathrm{sample}\;\mathrm{size}\;(1,116)}{\mathrm{Total}\;\mathrm{No}\;\mathrm{of}\;\mathrm{healthcare}\;\mathrm{workers}\;\mathrm{in}\;\mathrm{four}\;\mathrm{hospitals}} \end{array}$$

## Dependent and independent variables

• Compliance with standard precaution practice (Compliant/Non-compliant)

### Independent variables

• Socio demographic factors

Age of respondent, marital status, type of profession sex, work experience, and assigned place (ward).

• Institutional and behavioral factors

Availability of PPE, Presence of IP guidelines, availability of continuous running water supply, Training on infection prevention, attitude towards infection prevention practice, Knowledge of standard precautions, job satisfaction, and Chewing chat(khat) since most residents including healthcare workers in South Wollo zone practice consuming khat and this culture is rapidly expanding.

### Operational definition

**Compliance of standard precaution practice:** At the beginning of the analysis, those who reported that they were always compliant were taken as “compliant” and those who reported that they were sometimes and never compliant were taken as “non-compliant.” Next, the summation of the 22 compliance items was made. Then, the variable was recoded and dichotomized (compliant/non-compliant).

**Knowledge**: Knowledge of infection prevention and control practices was measured using 10 knowledge assessment questions. Each correct answer “yes” scored “1” and “no” scored “0” points for each knowledge question. If a HCW scored less than or equal to eight (≤80%) of all knowledge questions, it was considered to have poor knowledge (21).

**Attitude:** It was determined by computing the outcome of all 10 attitude assessment questions. Each criterion was given a value of 1 for agree and 0 for disagree. If a HCW scored less than or equal to eight (≤80%) of all attitude questions, it was considered to have a negative attitude (21).

**Continuous running water supply**: availability of continuously running water supply means that water is delivered continuously to every ward in need for 24 h a day, every day of the year.

### Data collection tools and quality assurance

A structured questionnaire was adapted from related literature (22–24). The questionnaire was first be prepared in English, translated to Amharic (the local language), and translated back to English to ensure consistency.

Training of the data collectors was provided by the principal investigator. It was taken two days focused on the questions in the survey instrument plus additional time for performing the observation of the practice and considering ethical issues. A pre-test was conducted using a 5% sample size of the total study sample in Woldeya hospital to establish the validity of the questionnaire. The questionnaire was amended based on the findings of the pre-test. The pre-test also served to familiarize the data collectors with the questionnaire and give them experience with data collection.

Six BSc nurses collected the data through a self-administered pretested and structured questionnaire. First, healthcare workers were asked to report each question about standard precautions and secondly, observation of certain practices during the time of data collection. Study participants were aware that they will be observed at some period, but they did not know exactly when they will be observed to reduce HCWs' behavior (Hawthorne effect). A single observer, who is also a co-investigator, was present at the study site to gather the data. The data were collected over a period of 30-minute sessions spread out across the day and night. If there was a privacy curtain drawn over the patient's bed, the normal precautionary measure was not followed. If the practice is carried out during times that are not designated by the WHO's recommended standard precaution practices, it is regarded as complementary and the observer did not keep track of it Three public health experts supervised the collection process for data quality control. The completeness and consistency of the questionnaires were checked daily before data entry.

### Statistical analysis

Data were entered using EpiData version 3.1 and exported to Statistical Package of Social Science (SPSS) version 25.0 for data cleaning and analysis. Once the data were entered, basic quality assurance measures including data cleaning using browsing of data tables after sorting, frequency distributions, cross-tabulations, summary statistics, and statistical outlier detection using sorting were performed. Descriptive statistics were used for categorical variables and mean ± SD (standard deviations) for continuous variables. Continuous variables were categorized using information from the literature, and categorical variables were re-categorized accordingly.

Bivariate [crude odds ratio (COR)] and multivariable [adjusted odds ratio (AOR)] values were calculated using logistic regression analysis with a 95% confidence interval [CI]. From the bivariate analysis, variables with *p* < 0.25 were considered as candidate variables for multivariable analysis. From the multivariable logistic regression analysis, variables with a significance level of *p* < 0.05 were taken as statistically significant and independently associated with compliance with standard precaution practice.

The presence of multi-collinearity among independent variables was checked using standard error at the cutoff value of 2. Model fitness was checked using the Hosmer-Lemeshow test which had a *p*-value > 0.05.

## Results

### Socio-demographic characteristics of the respondents

One thousand one hundred (98.6%) of the 1,116 healthcare professionals who participated in the study completed self-administered questionnaires. Regarding the gender of the study participants, 536 (50.7%) of the HCWs were married, and 620 (56.4%) of the participants were females. The majority of participants (657, or 59.7% of the total number of health care employees) were nurses, while approximately quarters (274, or 24.9%) were doctors. Seven hundred seventy-two respondents (70.2%) had bachelor's degrees, whereas 214 respondents (19.4%) had five to ten years of experience, and 826 respondents (75.1%) had more than ten years of work experience (Table 1).

**Table 1:** Socio-demographic characteristics of the health care worker in government hospitals of South Wollo zone, northeastern Ethiopia, 2021.

Variables	Category	Frequency	Percentage
Sex	Male	480	43.6
	Female	620	56.4
Age of respondent	19–30	218	19.8
	31–40	529	48.1
	41 and above	353	32.1
Marital status	Currently unmarried	564	51.3
	Currently married	536	48.7
Respondent working unit	Emergency room	74	6.7
	Pediatrics ward	101	9.2
	Delivery or gyn ward	183	16.6
	Medical ward	153	13.9
	Surgical ward	50	4.5
	Operation theater unit	95	8.6
	Laboratory	142	12.9
	Radiology unit	72	6.5
	Recovery	74	6.7
	OPD	118	10.8
	physiotherapy room	38	3.5
Educational status	Certificate	157	14.3
	Diploma	170	15.5
	BSc	448	40.7
	Medical doctor	241	21.9
	MSc/specialist	84	7.6
Work experience in years	<5 years	61	5.5
	5–10 years	213	19.4
	>10 years	826	75.1
Types of profession	Nurses	657	59.7
	Medical Doctor	274	24.9
	Laboratory professionals	88	8.0
	Other allied HCWs	81	7.4

### Institutional and behavioral factors

Only 222 (20.2%) of healthcare workers reported having a constant water supply for infection prevention practices, despite the fact that nearly three-quarters of 812 (73.8%) healthcare workers reported having an infection prevention guideline as a working protocol for preventing HCAIs.

More than seven in ten (77.9%) healthcare workers reported having personal protective equipment in their workplaces, and more than half (52.4%) of HCWs reported having completed training in infection prevention and control. However, 38.7% or more of the HCW population, or 426 people, still exhibited a negative attitude toward infection control measures. (Table 2).

**Table 2:** Institutional and behavioural factors of the healthcare worker in government hospitals of South Wollo zone, northeastern Ethiopia, 2021.

Variable	Category	Frequency	Percentage (%)
Availability of PPE	No	243	22.1
	Yes	857	77.9
Presence of IP guideline	No	288	26.2
	Yes	812	73.8
Availability of continuous water supply in the working unit	No	878	79.8
	Yes	222	20.2
Attitude toward infection prevention practices	Negative	426	38.7
	Positive	674	61.3
Training on infection prevention	No	524	47.6
	Yes	576	52.4
Knowledge on standard precautions	No	607	55.2
	Yes	493	44.8
Job satisfaction	No	880	80.0
	Yes	220	20.0
Chewing chat	No	927	84.3
	Yes	173	15.7

**Table 3:** Level of compliance with standard precautions among healthcare worker in government hospitals of South Wollo zone, northeastern Ethiopia, 2021.

Components of standard precautions (SPs)	Level of compliance		
	Never	Some times	Always
Wash hands before touching a patient	164 (14.9%)	592 (53.8%)	344 (31.3%)
Wash hands before clean or aseptic procedures	286 (26.0%)	230 (20.9%)	584 (53.2%)
Wash hands after body fluid exposure	28 (2.5%)	112 (10.2%)	960 (87.3%)
Wash hands after touching a patient	434 (39.5%)	324 (29.4%)	342 (31.1%)
Wash hands immediately after removal of gloves	45 (4.1%)	90 (8.2%)	965 (87.7%)
Wash hands between patient contact	513 (46.6%)	209 (19.0%)	378 (34.4%)
I protect myself against body fluids of all patients regardless of their diagnosis	465 (42.3%)	252 (22.9%)	383 (34.8%)
I provide nursing care considering all patients as potentially infectious	457 (41.5%)	256 (23.3%)	387 (35.2%)
Wash hands after touching patient surroundings	387 (35.2%)	327 (29.7%)	387 (35.2%)
I wear clean gloves whenever there is a possibility of exposure to any body fluids	24 (2.2%)	93 (8.4%)	983 (89.4%)
I change gloves between contacts with different patients	47 (4.3%)	82 (7.4%)	971 (88.3%)
I avoid wearing my gown out of hospital compounds	52 (4.7%)	405 (36.8%)	643 (58.5%)
I wear waterproof apron whenever there is a possibility of body fluid splashing in my body	50 (4.5%)	349 (31.7%)	701 (63.8%)
I wear eye goggles whenever there is a possibility of body fluid splashing in my face	484 (44.0%)	155 (14.1%)	461 (41.9%)
I sterilize all reusable equipment before being used on another patient	30 (2.7%)	133 (12.1%)	937 (85.3%)
I clean and disinfect equipment and environmental surfaces	153 (13.9%)	736 (66.9%)	211 (19.2%)
I segregate noninfectious wastes in black color coded dust bin	166 (15.1%)	454 (41.3%)	480 (43.6%)
I segregate infectious medical wastes in yellow colored coded dust bin	176 (16.0%)	383 (34.8%)	542 (49.3%)
I never bend needles with my hands	32 (2.9%)	157 (14.3%)	911 (82.8%)
I avoid removing used needles from disposable syringes	108 (9.8%)	364 (33.1%)	628 (57.1%)
I place used sharps in puncture-resistant container at point of use	20 (1.8%)	77 (7.0%)	1,003 (91.2)
I never recap needles	173 (15.7%)	275 (24.9%)	652 (59.3%)
Overall compliance with SPs	Non-compliant	889(80.8%)
	Compliant	211(19.2%)

**Table 4:** Proportion of HCWs compliance with standard precaution practice by professionals in government hospitals of northeastern Ethiopia, 2021.

Professions	Level of compliance with SPs			
	Compliant		Non-compliant	
	Frequency	Percentage	Frequency	Percentage
Nurses	114	10.4%	542	49.3%
Doctors	65	5.9%	209	19.0%
Laboratory	18	1.6%	70	6.3%
Other allied HCWs	14	1.3%	68	6.2%
Total	211	19.2%	889	80.8%

### Components of compliance measurement domains

We estimated a mean score of 30.38 ± 7.07 for the cumulative standard precautions compliance measuring domain, with a possible range of 0–44. Only one-third of them 344(31.3%), more than half 585(53.2%), and much more than three quarters 960(87.3%) of them always performed hand washing before handling a patient, before clean or aseptic operations, and after bodily fluid exposure respectively. Moreover, 342 (31.1%) of the HCWs practiced hand washing after making physical contact with a patient, 965 (87.7%) of HCWs washed their hands right after taking off their gloves, and 378 (34.4%) of HCWs applied hand washing in between patient contacts,.

**Figure 2:**
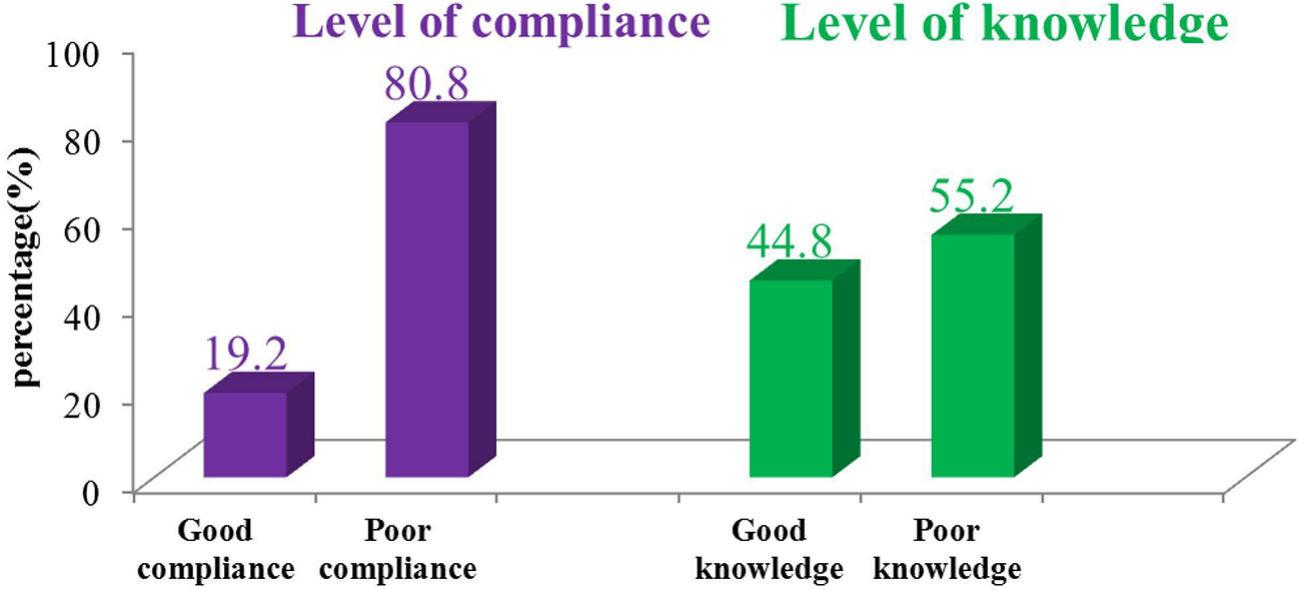
The magnitude of compliance with standard precaution practice and knowledge among healthcare workers in public hospitals of South Wollo zone, northeastern Ethiopia, April-May 2021 (*n* = 1,100).

About 35% of healthcare workers protected themselves against body fluids of all patient types regardless of their diagnosis and provided nursing care considering all patients as potentially infectious concurrently (Table 3). The overall proportion of HCWs who had good compliance with standard precaution practice was found to be 211(19.2%), 95% CI: 15.8–22.6) (Figure 2 and Table 4).

### Factors associated with standard precaution practices

Adjusted logistic regression analysis results indicated that work experience with less than 5 years (AOR = 2.51; 95% CI: 1.07–5.89), absence of continuous water supply (AOR = 2.24; 95% CI: 1.95–5.29), and negative attitudes toward infection prevention practices (AOR = 2.37; 95% CI: 1.17–4.79) were significantly associated with poor compliance of standard precautions practice (Table 5).

**Table 5:** Factors associated with standard precautions practice among healthcare workers in government hospitals of northeastern Ethiopia **.**

Variable	Category	Level of compliance		COR (95% CI)	AOR (95% CI)
		Non-compliant	Compliant		
Work experience	<5 years	41	20	1.42 (0.84–2.43)	2.51 (1.07–5.89)*
	5–10 years	180	34	2.37 (1.04–5.39)	0.56 (0.28–1.12)
	>10 years	668	157	1	1
Availability of PPE	No	158	85	0.80 (0.46–1.41)	1.28 (0.67–2.39)
	Yes	592	265	1	1
Availability of continuous water supply in working unit	No	632	245	1.73 (0.92–3.26)	2.24 (1.95–5.29)*
	Yes	117	106	1	1
Training on infection prevention	No	270	254	1.32 (0.84–2.07)	0.92 (0.47–1.79)
	Yes	479	97	1	1
Attitude towards infection prevention practices	Negative	216	209	1.88 (1.91–2.95)	2.37 (1.17–4.79)*
	Positive	533	142	1	1
Job satisfaction	No	619	261	1.40 (0.76–2.56)	1.09 (0.46–2.62)
	Yes	130	90	1	1
Chewing chat	No	668	283	1.39 (0.68–2.83)	1.07 (0.22–2.05)
	Yes	81	68	1	1

* indicates that the variable is statistically significant at *p* value <0.05.

## Discussion

Understanding reasons for non-compliance are the most important point to plan and to design interventions and strategies for improving behavior and programs that target conditions to prevent HCAI (6, 11, 25) and it is also critical to examine factors that influence compliance so that this study evaluated the compliance and determinant factors with conventional measures among 1,100 healthcare professionals in government hospital settings.

The best and most affordable method for preventing and controlling the transmission of HCAIs between patients and healthcare workers, patient attendants, and the general public has been thought to be HCW compliance with conventional precaution practices (6, 12, 14, 25).

The findings of this study showed that healthcare professionals generally adhered to standard precautions practices at a relatively low level. In contrast to earlier studies conducted in Gondar, Ethiopia (12%) (19), Hadiya, Ethiopia (15.0%) (26), Nigeria (14.85%) (27), and northeastern USA (17.4%) (28), the self-reported compliance of standard precaution practice among healthcare workers in this study was 19.2%.

The reasons for this non-adherence to SPs practice might be due to differences in a wide range of factors, including culture, economic and social factors, management commitment on the part of the institution, human resources, and individual factors like self-efficacy, beliefs, and knowledge of SPs measures, as well as professionals' lack of knowledge or resources.

The results of this study were, however, less favorable than those of studies carried out in Bahir Dar Town (41%) (29), Dawuro Zone, southwest Ethiopia (65%) (30), Hawassa Comprehensive Hospital, Sidama Ethiopia (56.5%) (31), North Shewa, Oromia (46.8%) (21), Addis Ababa, Ethiopia (66.1%), public hospitals in Abuja, Nigeria (76.2%) (32), and other two tertiary hospitals in Nigeria (50.8%) (33).

The primary factor contributing to the low rate of compliance with typical precautionary measures in this study might be a lack of training regarding their use and purpose in preventing HCAI (only half of HCWs reported that they received infection prevention training). Compliance with recommended safety measures can be improved *via* infection prevention training (34) and can reduce the perception of risk (35). Insufficient time, carelessness, discomfort, forgetfulness, lack of habit, and the belief that there is a low danger of infection may also contribute to limited compliance with routine precautionary measures.

The habit of hand washing practice in this study was a little higher than it was in previous studies done in comprehensive specialized hospitals in Gondar and Hawassa (19, 31). In those studies, it was found that hand washing was always practiced before touching a patient in Gondar (18.2%) and Hawassa (18.9%), between patient contacts in Gondar (19.4%) and Hawassa (27.1%). This difference might be brought on by varying workloads, a lack of hand washing facilities, difficulty accessing them, a lack of awareness of the need for hand hygiene in preventing infections, and a lack of a culture of safety that provides feedback.

The results of this study showed that HCWs’ compliance with recommended precautions was positively impacted by their job experience. In comparison to HCWs with less than five years of experience, those with more than ten years of experience were 2.51 times more likely to comply with standard precaution practices.

It is consistent with earlier findings from study conducted in Tanzania concerning, Egypt (36) compliance in segregation of non-sharp infectious waste from injection or blood draw (18.7%) (37), Abuja, Nigeria with mean compliance of standard precaution practice (23.2%) (32), Jordan (38) China (28.7%) (39), Shiraz, Iran (22.2%) and Italy (35).

Healthcare workers were 2.24 times more likely to be non-compliant with standard precaution measures than their counterparts if there was no continuous flowing water in the working department. A constant running water supply in the working area might directly promote compliance with standard precaution practices among HCWs because access to poor or interrupted water supply can hamper infection prevention practices in healthcare facilities. This association is consistent with the earlier studies done in Bahir Dar, Ethiopia (29), Hawassa, Ethiopia (31), Addis Ababa, Ethiopia (40) and Nigeria (33).

Healthcare workers with a negative attitude towards standard precaution practice were more than two times (AOR = 2.37) more likely to be non-compliant with standard precaution practice. This result is in line with studies done in Addis Ababa, Ethiopia (41) Shewa, Ethiopia (21) and Italy (35). This showed that having a positive attitude is essential for increasing compliance with infection prevention and control practices which may encourage healthcare workers to follow recommended precautions against the disease.

## Conclusions

Inadequate adherence to established precautionary procedures by healthcare professionals was found in South Wollo Zone government hospitals. The results of multivariable logistic regression analysis showed that lack of a continuous water supply, negative attitudes towards standard precaution practice, and work experience of less than five years were the key predictor variables for non-compliance with standard precaution practice.

The findings of this study highlight the urgent need for decision-makers to address inadequate compliance with basic precaution practices among HCWs in hospital settings. These findings should guide the development of efforts to promote the accessibility of a continuous water supply and alter the mindset of healthcare professionals through infection control training. We firmly urge national governments, the commercial sector, and the general public to pay close attention to healthcare worker safety.

## Limitations of the study

This study did not use a longitudinal follow-up design; instead, data were collected using a self-administered questionnaire and a single practice observation, raising the possibility of observer and responder bias. To ensure the quality of the data, we operationalized variables, followed the right procedures, and trained skilled data collectors.

## Data Availability

The raw data supporting the conclusions of this article will be made available by the authors, without undue reservation.
